# Selective Extraction of Entangled Textures via Adaptive PDE Transform

**DOI:** 10.1155/2012/958142

**Published:** 2012-01-16

**Authors:** Yang Wang, Guo-Wei Wei, Siyang Yang

**Affiliations:** ^1^Department of Mathematics, Michigan State University, East Lansing, MI 48824, USA; ^2^Department of Electrical and Computer Engineering, Michigan State University, East Lansing, MI 48824, USA

## Abstract

Texture and feature extraction is an important research area with a wide range of applications in science and technology. Selective extraction of entangled textures is a challenging task due to spatial entanglement, orientation mixing, and high-frequency overlapping. The partial differential equation (PDE) transform is an efficient method for functional mode decomposition. The present work introduces adaptive PDE transform algorithm to appropriately threshold the statistical variance of the local variation of functional modes. The proposed adaptive PDE transform is applied to the selective extraction of entangled textures. Successful separations of human face, clothes, background, natural landscape, text, forest, camouflaged sniper and neuron skeletons have validated the proposed method.

## 1. Introduction

Texture is one of the important features characterizing many natural and man-made images. Texture characterization and analysis are usually performed according to the spatial as well as frequency variations of brightness, pixel intensities, color, and texture orientation in the different regions of the image corresponding to different types of textures. For example, the roughness or bumpiness of an image usually refers to variations in the intensity values, or gray levels. Texture segmentation, recognition, and interpretation are critical for human visual perception and processing. As a result, research on texture analysis has received considerable attention in recent years. A large number of approaches has been proposed for texture classification and segmentation [1–16]. In general, texture analysis methods fall into two categories: statistical methods which analyze the Fourier power spectrum, gray level values, and various variance matrices of the input image, and structural methods which are knowledge-based algorithms with an emphasis on the structural primitives and their placement rules. Some examples of such methods include Markov random field models [17, 18], simultaneous autoregressive model [19], and fractal models [20]. Among many existing approaches, local variation minimization has been a popular and powerful technique in image analysis [21] with applications to the texture modeling [22]. Multiphase segmentation approaches are based on the structural division of gray scales [23]. More recently, multiresolution approaches have become more important in texture analysis [19, 24–26], where fixed-size neighborhood and window size are used to derive features at varying scales corresponding to the input image at different resolutions.

In general, the total texture extraction has become a mature technique in real applications. However, despite the progress in the past few decades, selective extraction of entangled textures encounters a number of difficulties. One difficulty is due to *spatial entanglement*, including orientation mixing of various textures. Another difficulty is due to *gray-scale entanglement*, especially the near-continuous merging of various textures. The other difficulty is due to *frequency entanglement* when two similar but different textures share overlapping frequency band in the frequency domain. This difficulty would especially plague texture analysis when many high-frequency textures coexist.

In this work, we propose an adaptive partial differential equation (PDE) transform approach for selective extraction of entangled textures. By using arbitrarily high-order PDEs, the PDE transform is able to decompose signals, images, and data into functional modes, which exhibit appropriate time-frequency localizations [27–31]. Additionally, the PDE transform is able to provide a perfect reconstruction. Unlike wavelet transform or Fourier transform, the PDE transform offers results in the physical domain, which enables straightforward mode analysis and secondary processing. Based on the image mode functions generated by the PDE transform method, the adaptive PDE transform algorithm calculates the variance of the local variation of the image mode functions followed by the corresponding thresholding analysis.

## 2. PDE Transform Method

In the past two decades, PDE-based image processing approaches have raised a strong interest in the image processing and applied mathematical communities and have opened new approaches for image denoising, enhancement, edge detection, restoration, segmentation, and so forth. The use of PDEs for image analysis started as early as 1980s when Witkin first introduced diffusion equation for image denoising [32]. The time evolution of an image under a diffusion operator is formally equivalent to the lowpass filter. After Perona and Malik introduced anisotropic diffusion equation in 1990 [33], nonlinear PDEs have found great applications for a variety of image processing tasks such as edge detection and denoising. Two important advances in the history of image processing, namely, the Perona-Malik equation and the total variation methods [21], employ second-order nonlinear PDEs for image analysis. The Willmore flow, proposed in 1920s, is a fourth-order geometric PDE and has also been used for surface analysis. In the past decade, fourth-order nonlinear PDEs have attracted much attention in image analysis [34–36].

Arbitrarily high-order nonlinear PDEs were introduced by Wei in 1999 to more efficiently remove image noise in edge-preserving image restoration [34]:

(1) $$\begin{array}{cc} u_{t}\left(r,t\right) & =\underset{q}{\sum }\nabla \cdot \left[d_{q}\left(u,\left|\nabla u\right|\right)\nabla \nabla^{2q}u\right]\\ & \;+e\left(u,\left|\nabla u\right|\right),\;\left(q=0,1,\ldots \right), \end{array}$$

where *u* ≡ *u*(**r**, *t*) is the image function, *d*_*q*_(*u*(**r**), |∇*u*(**r**)|, *t*) and *e*(*u*(**r**), |∇*u*(**r**)|, *t*) are edge-sensitive diffusion coefficients and enhancement operator, respectively. The Perona-Malik equation is recovered at *q* = 0 and *e*(*u*(**r**), |∇*u*(**r**)|, *t*) = 0. As in the original Perona-Malik equation, the hyperdiffusion coefficients *d*_*q*_(*u*(**r**), |∇*u*(**r**)|, *t*) in (1) can be chosen in many different ways. For instance, one can set

(2) $$\begin{array}{c} d_{q}\left(u\left(r\right),\left|\nabla u\left(r\right)\right|,t\right)=d_{q0}\exp\left[-\frac{{\left|\nabla u\right|}^{2}}{2\sigma_{q}^{2}}\right], \end{array}$$

where the values of constants *d*_*q*0_ depend on the noise level, and *σ*_0_ and *σ*_1_ are chosen as the local statistical variance of *u* and ∇*u*:

(3) $$\begin{array}{c} \sigma_{q}^{2}\left(r\right)=\bar{{\left|\nabla^{q}u-\bar{\nabla^{q}u}\right|}^{2}}\;\left(q=0,1\right). \end{array}$$

The notation $\bar{Y(r)}$ above denotes the local average of *Y*(**r**) centered at position **r**. In this algorithm, the statistical measure based on the variance is important for discriminating image edges from noise. As such, one can bypass the image preprocessing, that is, the convolution of the noise image with a test function or smooth mask.

In general, the nonlinear PDE operators described above serve as lowpass filters. PDE-based nonlinear highpass filters were introduced by Wei and Jia [37] in 2002. They constructed two weakly coupled PDEs to act as a highpass filter. Recently, this approach has been combined with Wei's earlier arbitrarily high-order nonlinear PDE operator to give [29]

(4) $$\begin{array}{c} \partial_{t}\left(\begin{array}{c} u_{m}\\ v_{n} \end{array}\right)=\left(\begin{array}{c} \overset{m-1}{\underset{j=0}{\sum }}\nabla \cdot d_{uj}\nabla \nabla^{2j}-\epsilon_{u_{m}},\epsilon_{v_{n}}\\ \epsilon_{u_{m}},\overset{n-1}{\underset{j=0}{\sum }}\nabla \cdot d_{vj}\nabla \nabla^{2j}-\epsilon_{v_{n}} \end{array}\right)\left(\begin{array}{c} u_{m}\\ v_{n} \end{array}\right), \end{array}$$

where *ϵ*_*u*_*m*__ ≡ *ϵ*_*u*_*m*__(|∇*u*_*m*_|) and *ϵ*_*u*_*n*__ ≡ *ϵ*_*u*_*n*__(|∇*v*_*n*_|) are made edge sensitive. As lowpass filters, both *d*_*uj*_ ≡ *d*_*uj*_(|∇*u*_*m*_|) ≥ 0 and *d*_*vj*_ ≡ *d*_*vj*_(|∇*v*_*n*_|) ≥ 0 when *j* is even. Similarly, both *d*_*uj*_(|∇*u*_*m*_|) ≤ 0 and *d*_*vj*_(|∇*u*_*m*_|) ≤ 0 when *j* is odd. We can define a PDE transform as

(5) $$\begin{array}{c} w_{m,n}\left(r,t\right)=u_{m}\left(r,t\right)-v_{n}\left(r,t\right)=H_{mn}\left(r,t\right)X\left(r\right), \end{array}$$

where *H*_*mn*_(**r**, *t*) can be regarded as a coupled nonlinear PDE operator. In order for (5) to work properly, we choose |*d*_*vj*_(|∇*v*_*n*_|)|≫|*d*_*uj*_(|∇*u*_*m*_|)|. As shown in our earlier work, by increasing the order of the highest derivative, one can increase frequency localization and accuracy of the PDE transform for mode decomposition [29]. The frequency selection of *w*_*m*,*n*_(**r**, *t*) also depends on the evolution time. High-order PDEs are integrated by using the Fourier pseudospectral method [29].

In the PDE transform, intrinsic mode functions *w* ^*k*^ are systematically extracted from residues *X* ^*k*^, that is,

(6) $$\begin{array}{c} w_{mn}^{k}=H_{mn}X_{mn}^{k},\;\forall k=1,2,\ldots , \end{array}$$

where *w*_*mn*_^*k*^ is the *k*th mode function. Here, the residue function is given by

(7) $$\begin{array}{c} X_{mn}^{k}=X_{mn}^{1}-\overset{k-1}{\underset{j=1}{\sum }}w_{mn}^{j},\;\forall k=2,3,\ldots , \end{array}$$

where *X*_*mn*_^1^ = *X*(**r**). Therefore, *X* = ∑_*j*=1_^*k*−1^ *w*_*mn*_^*j*^ + *X*_*mn*_^*k*^ is a perfect reconstruction of *X* in terms of all the mode functions and the last residue. The mode decomposition algorithm given in (6) is inherently nonlinear, even if a linear PDE operator might be used.

**Figure 1:**
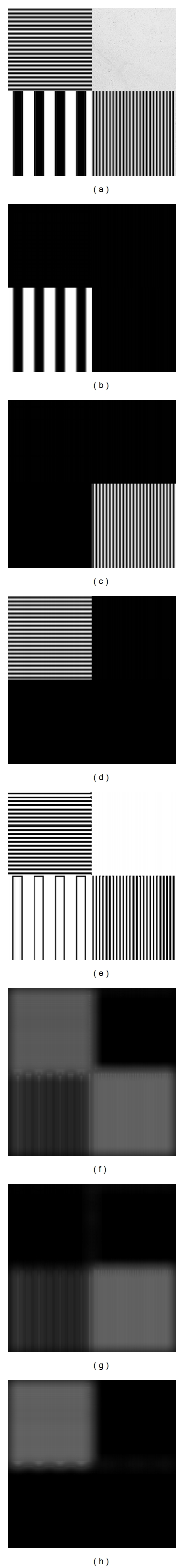
Extraction of various embedded textures using the PDE transform. (a) shows the original image composed of various horizontal and vertical textures. (b)–(d) show the three texture patterns extracted by applying the PDE transform, one at each time. (e) shows the edge mode obtained by applying the PDE transform to (a). (f) shows the variance of the local variation of the image mode function (e). (g) and (h) show the projection, or average, of the variance in (f) along *x*- and *y*-direction, respectively.

The PDE transform is applied to Figure 1(a) to extract the three textures in Figures 1(b), 1(c), and 1(d). Note that only one texture is isolated at each time, which means the proposed PDE transform is able to perform a controlled or selective segmentation of textures. The PDEs of up to order 200 have been used for the selective texture segmentation. Numerically, such high-order linear PDE needs to be solved in the frequency domain [29]. Due to the ideal frequency localization, three textures are separated with clear boundary sharpness.

## 3. Adaptive PDE Transform Algorithm

The separation of textures that are highly entangled in spatial locations, frequency ranges, and gray scales become a challenge, and conventional segmentation techniques are in general not applicable for such cases. For example, highly oscillatory textures can be separated from slowly varying background but cannot be separated from another texture with overlapping frequency distribution purely based on frequency fingerprints. To selectively distinguish such entangled textures of high frequency, one needs a mode decomposition algorithm that is able to be highly localized in frequency. Second-order PDEs are poorly localized in the frequency domain [29]. Whereas, the PDE transform with high-order PDEs provides desirable frequency localization [29]. However, the PDE transform by itself does not perform well for the separation of entangled textures. To this end, we introduce an adaptive PDE transform algorithm for selective texture extraction. The essence of the adaptive PDE algorithm lies in the realization that features of various textures are closely correlated with both the magnitude and smoothness of the gray-scale values, or, equivalently, the local variation of the image mode functions. Similar ideas have been implemented in other methods such as total variation [21].

Nonlinear PDEs have been widely applied to detect images with noises. However, despite better image edge protection, the nonlinear anisotropic diffusion operator may still break down when the gradient generated by noise is comparable to image edges and features [38]. Application of a preconvolution with a smoothing function to the image can practically alleviate the instability and reduce gray-scale oscillation, but the image quality is often degraded. One alternative solution introduced by Wei [34] is to statistically discriminate noise from image edges by a measure based on the local statistical variance of the image or its gradient. Such a local statistical variance based edge-stopping algorithm was found to work very well for image restoration.

**Figure 2:**
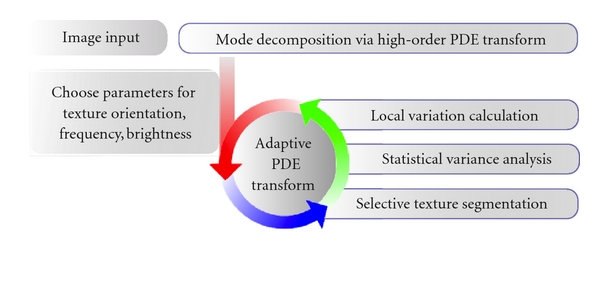
Algorithm of adaptive PDE transform for entangled texture separation.

Similar statistical analysis can be employed to perform selective texture extraction for images containing highly entangled and overlapping textures. In the present approach, we first compute the local variation of each pixel of the image mode functions obtained by the high-order PDE transform. Unlike the total variation, the local variation is still a function, of which the variance can be calculated:

(8) $$\begin{array}{c} E\left(X\left(r\right)\right)=\bar{{\left|\left|\nabla X^{k}\left(r\right)\right|-\bar{\left|\nabla X^{k}\left(r\right)\right|}\right|}^{2}}, \end{array}$$

where *X* ^*k*^(**r**) is the *k*th mode function obtained by the PDE transform (7), and |∇*X* ^*k*^(**r**)| is evaluated locally over the neighbor pixels. Equation (8) yields a statistical analysis which is used for various texture separation and segmentation with appropriate threshold values. Various threshold values need to be chosen to select the range of the variance corresponding to the particular texture of interest. All the previously classified textures are registered for sequential/recursive texture extractions. A flowchart of the adaptive algorithm of PDE transform is shown in Figure 2.

Figure 1(e) shows the edge mode obtained by applying the PDE transform to Figure 1(a).  Figure 1(f) shows the variance of the local variation of gray scale calculated using the adaptive PDE transform. Figures 1(g) and 1(h) show the projection, or average, of the variance in Figure 1(f) along *x*- and *y*-direction, respectively. By slicing out different domain of the variance in Figure 1(f), three different textures in Figures 1(b)– 1(d) are then perfectly separated from each other.

## 4. Applications

**Figure 3:**
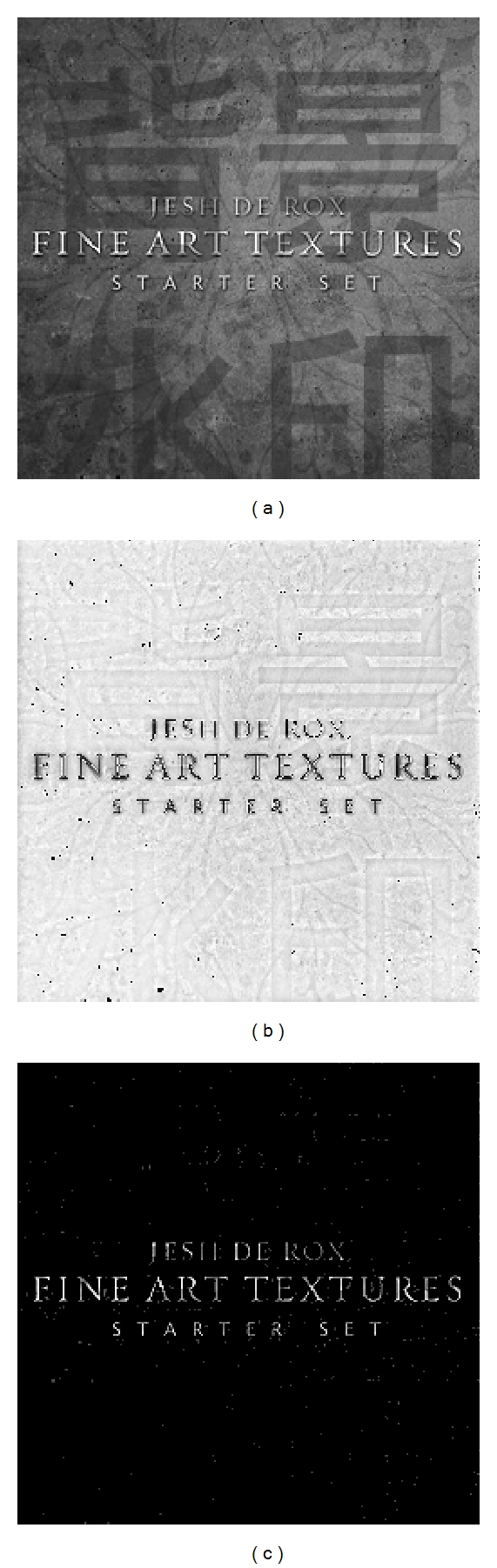
Extraction and separation of texts, background watermark, and textures of (a). Shown in the 3(b) and 3(c) are the image mode function and extracted texture using the proposed adaptive PDE transform.

In this section, the adaptive PDE transform is applied to three different cases to illustrate its superior capability of selective texture separation. The three images feature different types of entangled textures.  Figure 3(a) contains textures overlapping in the physical space with entangled frequency fingerprints. Figures 5(a) and 6(a) contain spatially segmented textures overlapping in the frequency domain.  Figure 7 contains textures with overlapping textures highly entangled in both the frequency and spatial domains.

### 4.1. Text-Image Separation

The adaptive PDE transform method employing the variance of the local variation of the image mode functions is applied to several benchmark test cases. In particular, separation of text and texture can be regarded as a generalized type of texture analysis. In Figure 3, texts of various fonts are imprinted on the background image. Additional background watermark in Chinese is also presented in Figure 3(a). The separation of English title from both background image and Chinese characters is a challenging task in terms of texture analysis because of the high degree of entanglement of very similar textures. Due to the font size difference in this application, high-order PDE transform plays an extremely important role in differentiating modes with slightly different frequency characteristics. In Figure 3(b), the PDE transform successfully suppresses the low-frequency parts and extracts the mode with frequency band mainly corresponding to texts. Such a procedure is similar to the edge detection in a general image processing. Statistical segmentation is then performed on the high-frequency mode. A suitable threshold value is used to cut off the region with low variance and yields only the texts as shown in Figure 3(c).

### 4.2. Selective Texture Extraction

**Figure 4:**
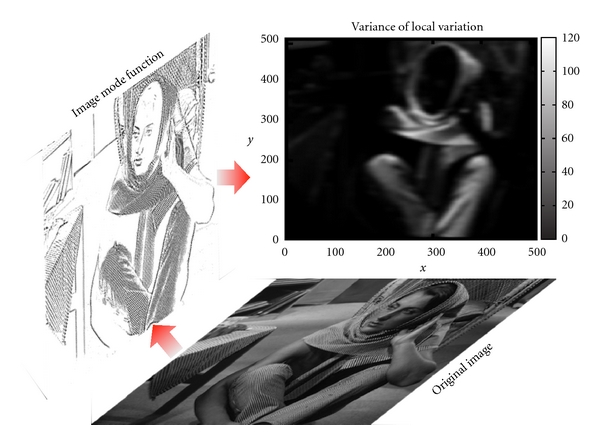
Adaptive PDE transform for selective texture extraction in the Barbara image. The variance of the local variation is shown in the top chart.

**Figure 5:**
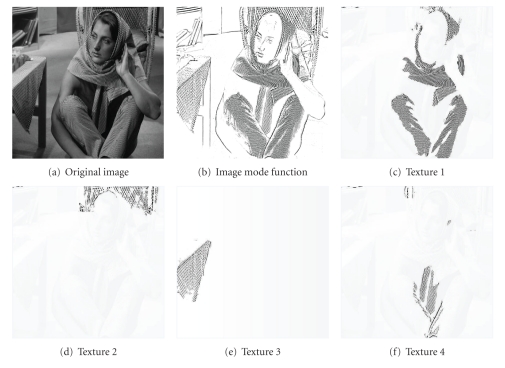
PDE transform is applied on (a) to extract edges of all textures into 5(b). Adaptive PDE transform is then applied to extract different textures from 5(b). In 5(c)–5(f), all the textures are superimposed on the original image for better viewing.

The present algorithm of selective texture extraction is also tested on one of the most widely used images, the Barbara, in Figure 5. Barbara image is a benchmark test for edge detection and denoising. It contains fine details of different textures such as the table cloth, curtain behind Barbara, scarf, and clothes on her. Distinctions between all these textures and the background are much larger than those among these textures, which leads to the difficulty of selective texture separation and segmentation. Due to the tiny difference between the frequency or spectrum features of different textures mentioned above, a highly frequency-selective separation method is required. However, the conventional Fourier method is not applicable for this case since the textures are entangled in the frequency domain. Moreover, conventional statistical segmentation approaches do not perform well for this case due to the gray-scale entanglement. The present adaptive PDE transform method performs well for the selective texture extraction in the Barbara image. The total texture, or image edge, is extracted from the high-frequency mode of the PDE transform as shown in Figure 5(b). The variance of the local variation is shown in Figure 4, which is calculated and employed for selective texture extraction and separation with appropriate thresholding values. The resulting textures are shown in Figures 5(c)–5(f) which correspond to those of clothes, curtain, and table cloth, respectively. The four textures in Figure 5 are superimposed on the original image for the purpose of a clearer visualization.

**Figure 6:**
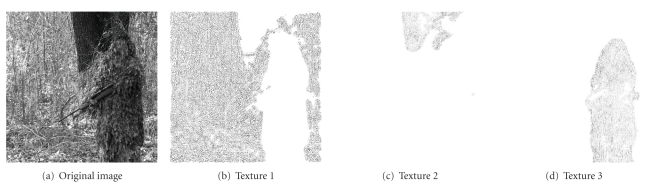
Sniper detection by using adaptive PDE transform method. Textures 1, 2, and 3 are, respectively, from the forest, the tree trunk, and the sniper.

In Figure 6, the present adaptive PDE transform is applied to detect a sniper hidden in the forest (Figure 6(a)). The whole image is composed of highly entangled textures. The boundaries between these textures are very challenging to be identified appropriately. In our approach, variance of the local variation is calculated and used for texture separation as in the previous examples. By appropriate thresholding, the variance can be decomposed into three regions corresponding to those of the forest, the tree trunk, and the sniper. The resulting texture modes are shown in Figures 6(b)–6(d).

### 4.3. Natural Neuron Skeleton Analysis

**Figure 7:**
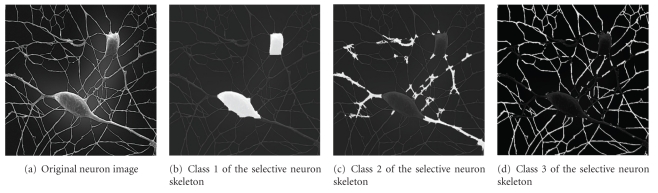
Neuron image classification by using the adaptive PDE transform.

In the previous introduction to the adaptive PDE transform algorithm and applications, local variation is defined and calculated for the intensity of image mode functions to selectively extract textures beyond the total texture extraction. The selective texture extraction can be generalized to indicate any spatial parts of the image characterized with specific (and usually functionally important) spatial orientation and/or frequency oscillation, such as different parts in the neuron synapses, brain cells, and retina vasculatures. In Figure 7(a), the image of a typical neuron is shown. With advanced imaging techniques made available, research scientists have been able to obtain more and clearer 2D images and 3D data of various neuron cells and networks, whose study will be important for identifying the relation between phenotype and genotype patterns in physiology and molecular biology. Closely related to the advancement in the experimental imaging techniques, various improved computational image processing techniques have been proposed to better analyze neuron images. Neuron morphology study has become more and more important since the shape and branching of dendrites in neurons are closely related to the structure and functioning of the neuron network. Advancements in both experimental imaging techniques and computational image enhancements have led to better visualization and exploration of neuron morphology [39–45]. In the study of neuron morphology, image processing and segmentation of cultured neuron skeletons provide details of how neuron grow and branches. In this work, we apply the adaptive PDE transform to the study of “natural” neuron skeleton to segment and classify neuron skeletons into desirable classes according to the spatial extension and frequency oscillation of neuron dendrites, very much like the way of dividing a total image texture into several selective fine textures. Such separation and classification enable secondary processing and analysis of neuron morphology, such as the computation of surface areas (for 2D images) or volumes (for 3D data) for different classes of neuron skeletons. Specifically, we aim to separate different parts, or textures, such as soma, dendrites, axon, terminal or lobe, and numerous ramifications, from the neuron imaging as shown in Figures 7(b)–7(d), where three classes of neuron parts are separated according to the spatial extension and frequency oscillation. Surface area of each class is listed in Table 1. Ratios of these surface areas and many other geometric ratios of neuron morphology are related, on both molecular and cellular levels, to the many physiological diseases as well as the classification of neuron synapses.

## 5. Conclusion

Selective extraction and separation of image textures involving spatial entanglement, gray-scale mixing, and high-frequency overlapping are challenging tasks in image analysis. In this work, we introduce an appropriate adaptation to our earlier partial differential equation (PDE) transform [29] to construct an adaptive PDE transform algorithm. The adaptation is realized via a proper thresholding with the statistical variance of the local variation of image functional mode functions. The present PDE transform enables one to decompose and separate modes with entanglement in both spatial and frequency domains. The proposed method is applied to several challenging benchmark images. Textures of very similar features in the same image are successfully decomposed and separated using the present adaptive PDE transform method.

## Figures and Tables

**Table 1:** Classification of natural neuron skeletons.

Neuron skeleton class	Physical meaning	Percentage of the total neuron surface area
Class 1 shown in Figure 7(b)	Soma (neuron cell body)	22%
Class 2 shown in Figure 7(c)	Major (root of) dendrite	24%
Class 3 shown in Figure 7(d)	Fine (tips of) dendrite	54%
